# Single port laparoscopic appendectomy: are we pursuing real advantages?

**DOI:** 10.1186/1749-7922-6-25

**Published:** 2011-08-10

**Authors:** Nereo Vettoretto, Vincenzo Mandalà

**Affiliations:** 1 Laparoscopic Surgery Unit, M. Mellini Hospital, Chiari (BS), Italy; 2 General and Emergency Surgery, Villa Sofia-Cervello joined Hospital, Palermo, Italy

**Keywords:** Laparoscopy, single port, appendectomy

## Abstract

Single port appendectomy, due to its cosmetic appeal and to a technique similar to classic laparoscopic appendectomy, is provoking an increasing number of publications and case series to explore its feasibility and effective improvements for patients with acute appendicitis. The margins for improvement are not so large, as laparoscopic appendectomy is, after 20 years from its beginning, still debated. A literature search has been accomplished to investigate the outcomes of the operation. 23 case series or retrospective comparisons with classic laparoscopy have been found. The numbers and low quality of the published data do not permit to draw evidence based conclusions. Still, trends seem to evidence an increase in complications especially in complicated appendicitis, which suggests caution in its dissemination outside clinical trials.

## Introduction

As soon as surgical access-natural orifice surgery (SA-NOS) has been clearly distinguished from endoscopical access-natural orifice surgery (EA-NOS), being the former more similar to classic laparoscopy and consequently more surgeon-friendly, the trend toward mini-invasiveness has caused a wide dissemination of single port-transumbilical surgical operations [1]. Single port appendectomy (SPA) is gaining quite an interest in the surgical community. Differently from single access cholecystectomy the operation is easily feasible and potentially safe, as the procedure can be carried out approximately in the same manner as the three-port laparoscopic appendectomy (LA)[2]. Some considerations, although, need to be pointed out, because the advantages for classical laparoscopic appendectomy are not quite ascertained as they are for laparoscopic cholecystectomy. Papers regarding SPA must be viewed in this particular scenario.

## Search method and results

A literature search has been made in PubMed and Google Scholar using key words "single port - single access - single incision AND appendectomy - appendicectomy - appendicitis", without language limits and excluding pediatric cases. Abstract selection was made on 157 papers, among which no randomized studies were found. 23 studies were pertinent with the review; 7 were pseudo-randomized retrospective case comparisons with LA (Oxford level of evidence 3b), and the remaining were case series (Oxford Level of evidence 4). The total number of SPA operations published is 589. Authors, years of publication, study designs and results are summarized in Table 1.

**Table 1:** list of studies published to june 30, 2011 regarding SPA

Author	Year	Type of study	Cases	Complications	Operative time (min)	Additional trocars used
**Barbaros **[26]	2010	Case series	3	none		none

**Bhatia **[2]	2011	Case series	17	none	63	none

**Budzynski **[27]	2011	Case series	2	none	25	y

**Chiu **[15]	2011	Case series	22	none	58	none

**Cho **[28]	2011	Case comparison with LA	23 (vs 20)	=	=	none

**Chow**[29]	2010	Case comparison with LA	40 (vs 33)		< (p < 0.05)	

**Chouillard **[30]	2010	Case series	41	3	39	none

**Dapri **[14]	2011	Case series	30	5	57	none

**Feinberg **[31]	2011	Case series	25	none	56	none

**Frutos **[32]	2011	Case series	73	none	40	none

**Hayashi **[19]	2010	Case series	1	none		none

**Hong**[33]	2009	Case series	31	3 (2 abscess, 1 omphalitis)	41	none

**Kim **[20]	2010	Case series	43	5	61	none

**Kang**[34]	2010	Case comparison with LA in complicated appendicitis	15	=		y

**Lee JA **[35]	2010	Case comparison with LA	35 (vs 37)	3 (2 wound infections, 1 abscess)	76	none

**Lee YS **[36]	2009	Case comparison with LA	72 (vs 108)	6	41	

**Nguyen **[37]	2009	Case series	1	none	40	none

**Raakow **[38]	2011	Case comparison with LA	20 (vs 20)	none	48	none

**Saber **[39]	2010	Case series	26	1 (omphalitis)	46	y

**Roberts **[40]	2009	Case series	13	none	87	none

**Teoh **[16]	2011	Case comparison with LA	30 (vs 60)	2 (1 abscess, 1 ileus)	=	

**Vidal **[17]	2011	Case series suprapubic approach	20	none	40	none

**Yu **[41]	2011	Case series suprapubic approach	6	none	48	none

**Total**			**589**	**28 (4.8%)**	**51**	

## Discussion

Clinical evidence and consensus development conferences have stated, so far, some evidence regarding the advantages of LA when compared to open appendectomy (OA)[3,4]. First of all, an utmost importance is given to patients' selection; in fact, grade A recommendation is advocated only for fertile women. The advantages in the remaining age/gender groups (elderly, men, obese, pregnant) are not so clear. Even in the case of complicated appendicitis (i.e. gangrene, abscess, generalized peritonitis and perforation) the laparoscopic approach carries doubts which are still unsolved, like the increase (although not always significant) in the post-operative intra-abdominal abscess' rate [5]. Indeed, overall complications are lowered, so as ileus and need for analgesics. Hospital stay, in-hospital costs, and return to work are subject to personal differences and are biased by unblinded randomization. The better cosmetics and patients' perceived quality of life tend to converge with OA in a long term follow-up, similarly to other disease treatments (i.e. colectomies) [6]. One thing is for sure: wound infections in LA are significantly and constantly less than in OA, even if OA is always less time-consuming [7]. As for the former, superficial wound infections are minor complications according to Clavien's classification, but they indeed heighten costs, outpatients' accesses and worsen quality of life in the first two-three weeks after the procedure [8]. Laparoscopic operative time is approximately 10 minutes longer (confidence interval 6-15 min) than the open operation, and this difference cannot influence significantly the outcome nor the economics [9]. A potential but unstudied further advantage could regard the rate of post-operative adhesions and that of incisional hernias. Some low grade evidence suggests that in certain age groups (younger and females) laparoscopy could lower the occurrence of small bowel obstruction and infertility in patients who undergo appendectomy [10].

These are key points in planning a comparative study between single port and three-port appendectomy. Factors involving operative time, length of hospital stay, analgesic requirement, improvement in cosmetics and port-site hernias have to be related to a substantial equivalence or lessening on morbidity and costs. Different devices have been approved for single access-multiport surgery. The oldest is the side-view 10 mm camera with a 3 mm operative channel used by gynaecologists. This system requires a 10 mm access, the very same as the usual umbilical optical access used in three port surgery; this modality did not gain popularity between general surgeons, due to the its absolute lack of triangulation for it generally requires a suspension for the appendix (trans-parietal stitches or supplemental miniport). The quality of view and the limited operability makes complicated appendicitis difficult to complete [11]. Anyway the so-called "video-assisted appendectomy", consisting in a mobilization and extraction of the organ via the single umbilical trocar, and subsequent open appendectomy, gained some popularity [12,13]. The first releases from the industry, beginning in the second half of the last decade, regarded multichannel ports, requiring a 1.5 to 2 cm incision of the fascia. They are disposable, have three-channels (usually two 5 mm and one 10/12 mm), recently broadened to 4-6 (due to the need for application to more complex operations), and generally require a longer 5 mm angulated camera. Instruments had to be redesigned to create an artificial triangulation by applying an articulation or a bending of the stalk: this implied a learning curve for the surgeon, who was obliged to a new cross-handed or left-handed dissection [14]. The conflict between the instruments and the camera remains a minor problem, differently from the initial single skin-incision associated to a three-port contiguous fascial entry adopting conventional trocars, which created instrumental and port-clashing and a substantial risk for incomplete fascial defect closing [15]. Moreover, the 5 mm camera does not offer the same view as the 10 mm camera, with consequent frequent blurring or dimming of the lens. Thus SPA finds its ideal application in uninflamed or poorly inflamed appendicites, especially during the learning curve: a case-controlled comparative study evidences a higher rate of re-interventions in case of complicated appendicitis treated in single access [16]. Regarding wound infection, some of these multiport devices have to be removed together with the appendix, thus permitting a contact between the inflamed organ and the abdominal wall. In the few published case comparisons we cannot evidence an increase in the suppuration rate if compared to classic laparoscopy, but this data is likely to grow if studied in larger series, especially if that kind of port is used [17]. Indeed, if we sum the overall complications of the published SPA cases (including intraabdominal abscesses, omphalites, ileus, either medically or surgically treated) we find a 4.8% rate of surgical complications, which is higher than that reported in the literature for LA. The use of dedicated instruments might rise the cost of single port appendectomy; this problem has been overcome with difficulty in the era of LA (only recently cost analyses have shown a similar cost compared with OA), and SPA might induce the surgeon, once again, to increase the utilization of high-tech instruments (i.e. radiofrequency or ultrasonic scalpels for dissection, staplers for the stump) to enhance safety and to lower operative time [16]. These devices should be utilized only in more complex procedures, like colonic resections or other major abdominal one-port surgeries, which will probably be an ideal application, in the future, for robotic single-site platforms [18]. Home-made devices built with a low-cost surgical glove have been proposed as less-costly alternatives to dedicated multichannel trocars [19]. Single port operation doesn't seem more time consuming than classical laparoscopy, differently from cholecystectomy, thanks to the easy exposure of the organ; the mean time reported for SPA in our summary is 51 minutes. Time-saving results (evidenced in some studies) do have to be confirmed by larger trials [11]. With regard to cosmetics, two approaches have been studied in SPA: trans-umbilical and supra-pubic [20,18]. Both seem safe and permit a good visualization of the surgical field. In the former the scar in deepened in the umbilical scar, and in the latter it is covered by pubic hair. Patient's satisfaction has still to be tested in larger cohorts, but the first studies regarding quality of life in single port cholecystectomy do not seem to evidence significant improvements in comparison to four port-laparoscopy [21]. In classical LA care should be taken in order to place the trocar incisions parallel to Langers' lines of wound healing [22]; moreover 10/12 operative trocar (if used) should be put preferably in the supra-pubic area (instead of left or right flank). Whenever possible 5 mm trocars should be preferred, at least in those cases in which the appendix can be extracted from the optical trocar. Alternative supra-pubic positions have been described in order to improve cosmetics [23]. The use of miniports (minilaparoscopic appendectomy) has been shown to carry similar results with less analgesic requirement and rate of conversion in non-complicated cases [24]. These tricks might render the difference between single trocar and classic laparoscopy not influential in terms of visible scars. Another claimed advantage regards incisional hernias. This problem increases in the lower abdomen, where the intra-abdominal pressure is higher in the upstanding position. The rationale for larger incisions of the fascia, required for single trocar access, is that the "open" technique is mandatory, and so is the closure suture (under direct vision): this should lower the incisional hernias. This isn't anyway proved by trials in the literature, where different trocar entries are never studied in association with postoperative observation of port-site hernias. If this hypothesis should be ever demonstrated "open access" (using Hasson technique) should be routinely performed for the induction of pneumoperitoneum also in conventional laparoscopy.

## Conclusions

In conclusion, single port appendectomy is technically feasible for most cases of appendicitis. Anyway, the possible advantages, advocated for single access surgery in other diseases, should be carefully considered in relation to the advantages of laparoscopic appendectomy over the open appendectomy, which are not so evident even after more than twenty years from the first operation by Hans de Kok [25]. Therefore, on the basis of the published results of this technique, we recommend its application only to restricted groups of patients: notably pre-menopausal women in which, after explorative laparoscopy (10 mm trocar passed through an intra-umbilical incision), the level of inflammation of the appendix is not so high and absolutely not complicated by generalized peritonitis, abscess, gangrene or perforation; if these conditions are satisfied, the 10 mm trocar can be substituted with a multi-port single trocar which should guarantee a complete wound protection during the extraction of the organ. Trials should be addressed to evidence the effective improvements related to SPA, but in the meanwhile the dissemination of the technique should be carefully addressed, for the higher costs related to dedicated instruments and devices should be justified by concomitant ameliorations in the operative and post-operative patients' quality of life.

## List of abbreviations

LA: laparoscopic appendectomy; OA: open appendectomy; SPA: single port appendectomy.

## Competing interests

The authors declare that they have no competing interests.

## Authors' contributions

NV had the idea for the review and made the literature research and the writing of the article, VM has been involved in the drafting of the manuscript, revision, interpretation of the data and critical appraisal of the study. All authors read and approved the final manuscript.
